# Inorganic Arsenic–Related Changes in the Stromal Tumor Microenvironment in a Prostate Cancer Cell–Conditioned Media Model

**DOI:** 10.1289/ehp.1510090

**Published:** 2015-11-20

**Authors:** Joseph J. Shearer, Eric A. Wold, Charles S. Umbaugh, Cheryl F. Lichti, Carol L. Nilsson, Marxa L. Figueiredo

**Affiliations:** Department of Pharmacology and Toxicology, University of Texas Medical Branch, Galveston, Texas, USA

## Abstract

**Background:**

The tumor microenvironment plays an important role in the progression of cancer by mediating stromal–epithelial paracrine signaling, which can aberrantly modulate cellular proliferation and tumorigenesis. Exposure to environmental toxicants, such as inorganic arsenic (iAs), has also been implicated in the progression of prostate cancer.

**Objective:**

The role of iAs exposure in stromal signaling in the tumor microenvironment has been largely unexplored. Our objective was to elucidate molecular mechanisms of iAs-induced changes to stromal signaling by an enriched prostate tumor microenvironment cell population, adipose-derived mesenchymal stem/stromal cells (ASCs).

**Results:**

ASC-conditioned media (CM) collected after 1 week of iAs exposure increased prostate cancer cell viability, whereas CM from ASCs that received no iAs exposure decreased cell viability. Cytokine array analysis suggested changes to cytokine signaling associated with iAs exposure. Subsequent proteomic analysis suggested a concentration-dependent alteration to the HMOX1/THBS1/TGFβ signaling pathway by iAs. These results were validated by quantitative reverse transcriptase–polymerase chain reaction (RT-PCR) and Western blotting, confirming a concentration-dependent increase in HMOX1 and a decrease in THBS1 expression in ASC following iAs exposure. Subsequently, we used a TGFβ pathway reporter construct to confirm a decrease in stromal TGFβ signaling in ASC following iAs exposure.

**Conclusions:**

Our results suggest a concentration-dependent alteration of stromal signaling: specifically, attenuation of stromal-mediated TGFβ signaling following exposure to iAs. Our results indicate iAs may enhance prostate cancer cell viability through a previously unreported stromal-based mechanism. These findings indicate that the stroma may mediate the effects of iAs in tumor progression, which may have future therapeutic implications.

**Citation:**

Shearer JJ, Wold EA, Umbaugh CS, Lichti CF, Nilsson CL, Figueiredo ML. 2016. Inorganic arsenic–related changes in the stromal tumor microenvironment in a prostate cancer cell–conditioned media model. Environ Health Perspect 124:1009–1015; http://dx.doi.org/10.1289/ehp.1510090

## Introduction

Inorganic arsenic (iAs) is a ubiquitously distributed environmental toxicant that is classified as a Group 1 carcinogen by the International Agency for Research on Cancer (IARC) (IARC 2012). The deleterious effects of iAs exposure on human health have been observed for thousands of years (Bolt 2012) and continue to be of great concern today. Recent evidence estimates that nearly 137 million people worldwide are exposed to levels of iAs in their drinking water that exceed the recommended safety limits of 10 ppb mandated by the U.S. Environmental Protection Agency (EPA) (U.S. EPA 2001; Ravenscroft 2007).

The results of both epidemiological and experimental studies have suggested a direct link between iAs exposure and prostate cancer (García-Esquinas et al. 2013; Lewis et al. 1999; Prins 2008; Tokar et al. 2010; Treas et al. 2013). The most prevalent and second-deadliest form of cancer in men [American Cancer Society (ACS) 2015)], prostate cancer costs the U.S. health care system nearly 10 billion dollars annually (Roehrborn and Black 2011). Unfortunately, the propensity for prostate cancer to become resistant to conventional chemotherapeutics by losing sensitivity to androgen ablation therapy makes it very difficult to treat (Feldman and Feldman 2001) in part because prostate cancer often produces secondary bone metastases, which are associated with poor survival (Ye et al. 2007). Thus, determining whether environmental exposures play a role in the etiology of prostate cancer may reduce the associated health burden by preventing malignant tumor progression.

Although the causal relationship between iAs exposure and cancer is well established (IARC 2012), the precise mechanism(s) underlying iAs-induced carcinogenesis have yet to be fully characterized. Several studies have suggested production of reactive oxygen species (Pi et al. 2002), genome instability (Sciandrello et al. 2004), and aberrant expression of DNA repair machinery (Martinez et al. 2011) as potential mechanisms. However, many of these epithelial-centric hypotheses have not explored the potential contribution of stromal cells within the tumor microenvironment in mediating the tumorigenic effects of iAs.

The tumor microenvironment consists of a variety of biologically active stromal cells, which dynamically secrete a wide spectrum of cytokines and growth factors (Witz 2008). Many of these released paracrine factors have been associated with the progression of prostate cancer (Chung et al. 2005). The prostate possesses a unique microenvironment that includes the periprostatic adipose tissue layer, whose increased thickness has been tied to prostate cancer severity and poor prognosis (Finley et al. 2009; Ribeiro et al. 2012b; van Roermund et al. 2011). One specific cell population that is enriched in the prostate microenvironment is adipose-derived mesenchymal/stromal stem cells (ASCs) (Ribeiro et al. 2012c).

Several reports have established that mesenchymal stem cells (MSCs), including ASCs, possess a tropism towards areas of increased inflammation such as tumors (Spaeth et al. 2008; Zolochevska et al. 2012). It is thought that these cell types are recruited into tumors to promote healing and to potentially quell the inflammatory response (Aggarwal and Pittenger 2005; González et al. 2009). Although many investigators, including ourselves, are utilizing ASCs as a potential therapeutic modality because of their inherent tropism towards tumors (Hall et al. 2007; Zolochevska et al. 2014), emerging evidence has suggested that endogenous ASCs may also play a critical role in tumor progression depending on the cellular context (Barron and Rowley 2012; Kidd et al. 2012). Moreover, the potential effects of environmental toxicants such as iAs on these endogenous cells is unknown. We hypothesized that exposure of a stromal cell population enriched in prostate tumors (ASCs) to iAs would modify their cell-cell communication with epithelial tumor cells. Therefore, in our experimental design, our goal was to establish *a*) whether changes in cell–cell communication between ASCs and prostate cancer cells following exposure to iAs can be detected; *b*) whether potential mechanisms for the iAs-mediated ASC alterations can be identified, and *c*) whether *in vitro* data can be used to assess how iAs-mediated changes in ASCs might enhance prostate cancer progression.

To the best of our knowledge, our results indicate for the first time that exposure to iAs alters stromal–epithelial signaling by modulating global cytokine signaling in a population enriched in the prostate tumor microenvironment (ASCs), which may explain the enhanced effects on prostate cancer cell viability. Specifically, we have identified that exposure of ASCs to iAs aberrantly modulates TGFβ signaling, a pathway strongly linked with the regulation of prostate cancer progression (Li et al. 2008; Santamaria-Martínez et al. 2009). We propose that potential rewiring or reprogramming of stromal cells within a tumor can culminate in an environment that is more suitable for the progression of prostate cancer, thus identifying a novel mechanism by which iAs exposure may elicit its carcinogenic effects.

## Materials and Methods

### Cell Culture and iAs Exposure

The human prostate cancer cell line PC3 was obtained from American Type Culture Collection and maintained in 10% fetal bovine serum (FBS, Fisher) in Roswell Park Memorial Institute medium 1640 (RPMI, Corning). ASCs from two male Caucasian donors, generously provided by J. Gimble (Tulane University), were collected as previously described (Zolochevska et al. 2014) and cultured on fibronectin-coated plasticware in modified media conditions that have been described previously (Ruiz et al. 2010). ASCs from Donor 1 were isolated from midsection liposuction, and Donor 2 ASCs were isolated from liposuction samples from breast tissue. Briefly, “ASC media” was prepared to a final concentration of 60% Dulbecco’s Modified Eagle Medium (DMEM, Corning), 40% MCDB-201 medium (Sigma), 5% FBS, 1× insulin–transferrin–selenium (Corning), 1 nM dexamethasone (Acros Organics), 10 ng/mL epidermal growth factor (eBioscience), 0.1 μM ascorbic acid (Acros Organics), and 1× antibiotic–antimycotic (Gibco). For iAs exposure, culture medium with 0, 1, 10, or 75 ppb iAs (sodium arsenite, RICCA) was used. All ASC experiments used cells with a passage number < 10.

### iAs Cytotoxicity

One thousand ASCs were seeded per well in a 96-well plate format in 100 μL ASC medium. Baseline cell viability was determined after 24 hr by adding 10 μL of CCK-8 (Cell Counting Kit-8, Dojindo) and incubating for 2 hr at 37°C. CCK-8 is a water-soluble tetrazolium salt that upon entry into a live cell is rapidly reduced by intracellular dehydrogenases to form a spectroscopically active formazan compound, the amount of which is proportional to the number of live cells present (Ishiyama et al. 1997). Absorbance was read at 450 nm using a Glomax plate reader (Promega). Media were replaced with ASC media containing varying amounts of iAs and reassessed by CCK-8 assay after 48 hr.

### ASC Cell Culture Conditioned Media (CM) and PC3 Coculture

ASCs were exposed to 0 or 75 ppb iAs ASC media for 1 week as described above. ASCs were washed to remove residual iAs, and the media were exchanged to a low-serum media [2% FBS DMEM:F12 (Corning)] and incubated without the presence of iAs for an additional 24 hr. CM was collected and stored at –80°C. PC3 cells were seeded at a density of 2,000 cells/well in a 96-well plate format and were allowed to adhere for 24 hr. Cell viability was determined (CCK-8), and media were replaced with 2% FBS DMEM:F12 (control), or 2% FBS DMEM:F12 containing CM from ASCs that were previously exposed to 0 or 75 ppb of iAs, reflecting a 1% ASC proportion relative to PC3 cells. Cell viability was reassessed by CCK-8 after 48 and 96 hr.

### Global Cytokine Expression Array

Briefly, ASCs exposed to 1 week of 0, 1, 10, or 75 ppb iAs were collected. The results were analyzed using a Bio-Plex Pro™ Human Cytokine 27-plex Assay (Bio-Rad) according to the manufacturer’s instructions. Cytokine concentrations were determined using the Bio-Plex system.

### Nontargeted Proteomic Analysis and Bioinformatics

Sample preparation and nano-liquid chromatography tandem mass spectrometry (nanoLC-MS/MS) analysis for nontargeted proteomics was performed as previously described (Lichti et al. 2014), with minor modifications. Data files were analyzed as previously described (Lichti et al. 2014). Briefly, automatic and manual alignment of mass-to-charge ratio (*m/z*) and retention time were performed using Progenesis QI for Proteomics (v.18.214.1528, Nonlinear Dynamics), and the top five spectra for each feature were exported for database searching in PEAKS (Han et al. 2005; Ma et al. 2003; Zhang et al. 2012) (v.6, Bioinformatics Solutions Inc.) and Mascot (v.2.3.02, Matrix Science) against the UniprotKB/SwissProt Human database (June 2014 version, containing 20,213 proteins) appended with the Common Repository of Adventitious Proteins contaminant database (2012.01.01 version, http://www.thegpm.org/crap/). After importing the resulting peptide–spectrum matches (95% peptide probability) into Progenesis QI, normalized peptide intensity data for unique peptides were exported and filtered to remove methionine-containing peptides (Perrin et al. 2013) and all peptide modifications except for cysteine carbamidomethylation. Peptide intensities were imported into DanteR (v.0.1.1) (Karpievitch et al. 2009; Polpitiya et al. 2008) and processed for protein quantification as previously described (Lichti et al. 2014). The resulting proteins were assessed for statistical significance between groups by a two-way analysis of variance (ANOVA) with ASC donor and iAs dosage as factors, and *p-*values were corrected for multiple testing (Benjamini and Hochberg 1995).

### Ingenuity Pathway Analysis

Ingenuity pathway analysis (IPA) (Ingenuity Systems, v.18488943, build 308606M, created 23 June 2014, http://www.ingenuity.com) was used to analyze proteomics datasets containing protein identifiers, fold changes, and *p-*values from two-way ANOVAs comparing ASCs from both donors exposed to either 0 or 75 ppb iAs for 1 week. Predicted network analysis was performed using proteins with a *p*-value < 0.05.

### Quantitative Reverse-Transcriptase Polymerase Chain Reaction

Total RNA was isolated using the RNeasy Mini Kit (Qiagen). One microgram RNA was reverse-transcribed using *amfiRivert* Platinum cDNA Synthesis Master Mix (GenDEPOT). Quantitative reverse-transcriptase polymerase chain reaction (qRT-PCR reactions contained 1 μL cDNA template, 2× SYBR Green Master Mix (Applied Biosystems), and 10 mM forward and reverse primers for both experimental and β-actin controls. qRT-PCR was performed on an Eppendorf Realplex 2S Mastercycler® (Eppendorf) using the following conditions: 40× at 95°C for 3 min, 95°C for 3 sec, 60°C for 30 sec, and 72°C 8 sec. Analysis was performed using EP Realplex software (v.2.2, Eppendorf).

### Western Blot Analysis

Total proteins were isolated using RIPA Buffer (Thermo Scientific) and freeze-thawing at –80°C in the presence of 1× Halt Protease Inhibitor (Thermo Scientific), 5 mM ethylenediaminetetraacetic acid (EDTA), and Phosphatase Inhibitor Cocktails 2 and 3 (Sigma-Aldrich). Protein concentration was determined using a BCA Protein Assay (Thermo Scientific), and 50 μg protein was separated via electrophoresis on a Bolt 4–12% Bis-Tris Plus Gel (Life Technologies) and transferred onto membranes using the Iblot2 (Life Technologies) system, according to the manufacturer’s instructions. Immunoblotting was performed using antibodies for heme oxygenase-1 (HMOX1) (catalog no. 13248, Abcam; 1:250) and thrombospondin-1 (THBS1) (catalog no. 1823, Abcam; 1:1,000) with α-mouse secondary (catalog no. 925-32210, LICOR; 1:15,000). β-Actin was probed using a 1°-horseradish peroxidase (HRP) antibody (MA5-15739, Thermo Scientific; 1:1,000) and developed using ECL Plus (Thermo Scientific). Detection of blots was performed using a LICOR Odyssey LC system and quantified using Image Studio (v.4.0, LICOR).

### TGFβ Luciferase Reporter Assay

ASCs were seeded at a density of 10,000 cells/well in a 96-well plate and incubated for 24 hr. Cells were transfected with XFECT transfection reagent (Clontech) using 1 μg total DNA per sample optimized per the manufacturers’ instructions. Briefly, cells were transfected using 500 ng of an SBE-4-Luc plasmid (catalog no. 16495, Addgene), 500 ng validated short hairpin RNAs (shRNAs)(SHC002, Sigma-Aldrich), and 10 ng cytomegalovirus (CMV)-LacZ (β-Gal) plasmid for 6 hr. Media were replaced with fresh 2% FBS DMEM:F12 with 0, 1, 10, or 75 ppb iAs, and cells were allowed to incubate for 48 hr. Cells were collected in 1× Passive Lysis Buffer (Promega), and luciferase expression was detected using the Luciferase Assay System (Promega) and a Glomax 96-well luminometer (Promega).

### Statistical Analysis

Unless otherwise indicated, all assays were run in triplicate with values shown as the mean ± SEM or 95% confidence interval (CI). Student’s *t*-test was used for pairwise analysis, and one- or two-way ANOVA was used for comparisons across groups. *p*-Values < 0.05 were considered to be statistically significant.

## Results

### iAs-Induced ASC Cytotoxicity

**Figure 1:**
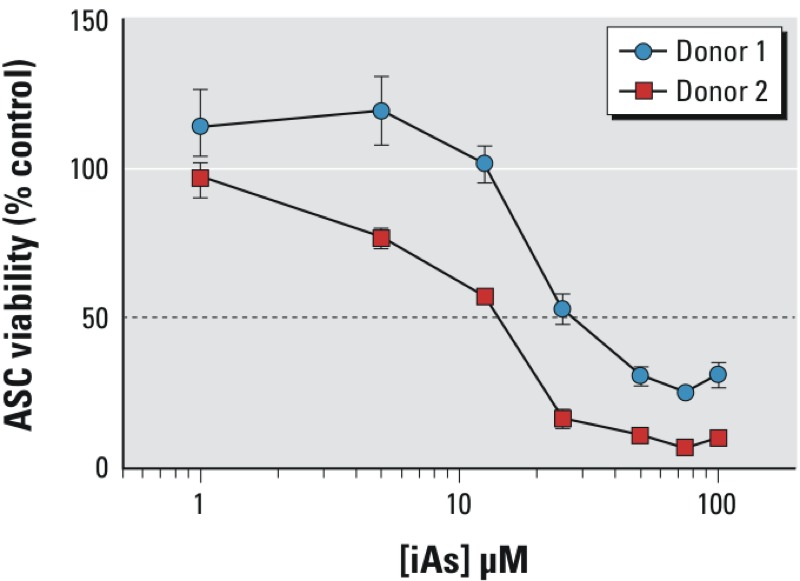
ASC viability after iAs exposure. Graph represents ASC (adipose-derived mesenchymal/stromal cell) viability after 48 hr of inorganic arsenic (iAs) exposure for two biological ASC donors. The results are displayed as the mean ± SEM (*n* = 3) normalized to percent control (0 ppb) for each individual donor. The dotted line represents the IC50.

ASCs were examined to elucidate the effects of iAs exposure on stromal cell–cell communication. To determine the cytotoxic effects of iAs exposure, we exposed naïve primary ASCs (from earlier than passage 10) from each donor to concentrations of iAs ranging from 1–100 μM (corresponding to 75–7,500 ppb) for 48 hr. Subsequent cell viability was measured by CCK-8 analysis as described in “Materials and Methods.” IC50 values (Figure 1) demonstrated some biological variability in iAs sensitivity between donors IC50_Donor1_ = 19.3 μM (95% CI: 15.5, 24.1 μM); IC50_Donor2_ = 12.8 μM (95% CI: 10.5, 15.7 μM). Based on results from the ASC viability assay and from the literature (Ravenscroft 2007), we chose to employ three exposure levels of iAs for subsequent experiments. The exposures chosen were 1, 10, and 75 ppb to appropriately reflect sub–U.S. EPA, U.S. EPA, and super–U.S. EPA levels, respectively (U.S. EPA 2001). Additionally, these exposures represented nonlethal concentrations of iAs that reflect the environment in which humans are exposed.

### Assessment of Prostate Cancer Cell Viability in Co-culture with iAs-Exposed CM

**Figure 2:**
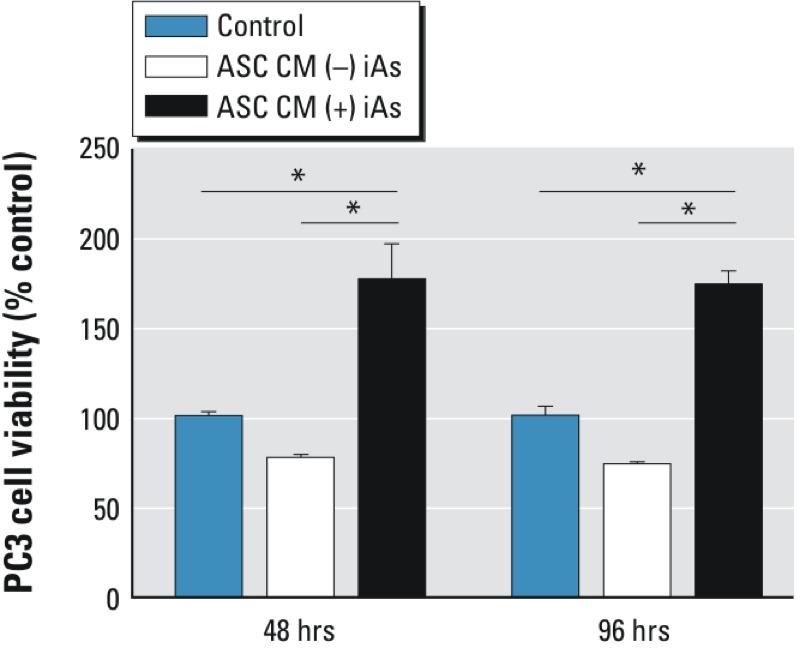
iAs-exposed ASC CM alters PC3 cell viability. 0- or 75-ppb inorganic arsenic (iAs)-exposed conditioned media (CM) were collected from adipose-derived mesenchymal/stromal cells (ASCs) after 1 week of exposure and applied to PC3 cells. The graph shows PC3 cell viability after 48 and 96 hr compared with cells exposed to control media [2% fetal bovine serum (FBS) Dulbecco’s modified Eagle medium (DMEM):F12 media]. The results are displayed as the mean ± SEM (*n* = 3). Pairwise comparison was performed using Student’s *t*-test with a *p*-value < 0.05 (*) considered significant.

We next examined the effects of ASC CM on PC3 viability. ASCs were exposed to either 0 or 75 ppb iAs for 1 week, after which CM was collected. The 1-week time point was chosen because previous reports have suggested that following chronic exposure, arsenic metabolites reach equilibrium in the urine of exposed human individuals in approximately 1 week (Buchet et al. 1981), reflecting a steady-state exposure. We designed our CM studies to contain 1% CM from ASCs relative to cultured PC3 cells, reflecting a biologically relevant concentration of MSCs localized within the prostate microenvironment (Brennen et al. 2013). Following exposure of 0- or 75-ppb iAs-treated 1% CM, PC3 cell viability was determined at 48 and 96 hr. Our results suggested that CM from ASCs exposed to iAs for 1 week increased prostate cancer cell viability at both 48 and 96 hr (176 ± 21%, *p* = 0.022 and 173 ± 11%, *p* = 0.003, respectively) (Figure 2, black bars) compared with 2% FBS DMEM:F12 media alone. These results suggest that exposure to iAs may alter ASCs in a manner that modifies how the stroma communicates with prostate tumor cells by augmenting tumor cell viability.

### Effects of iAs Exposure on Global Cytokine Expression by ASCs

**Figure 3:**
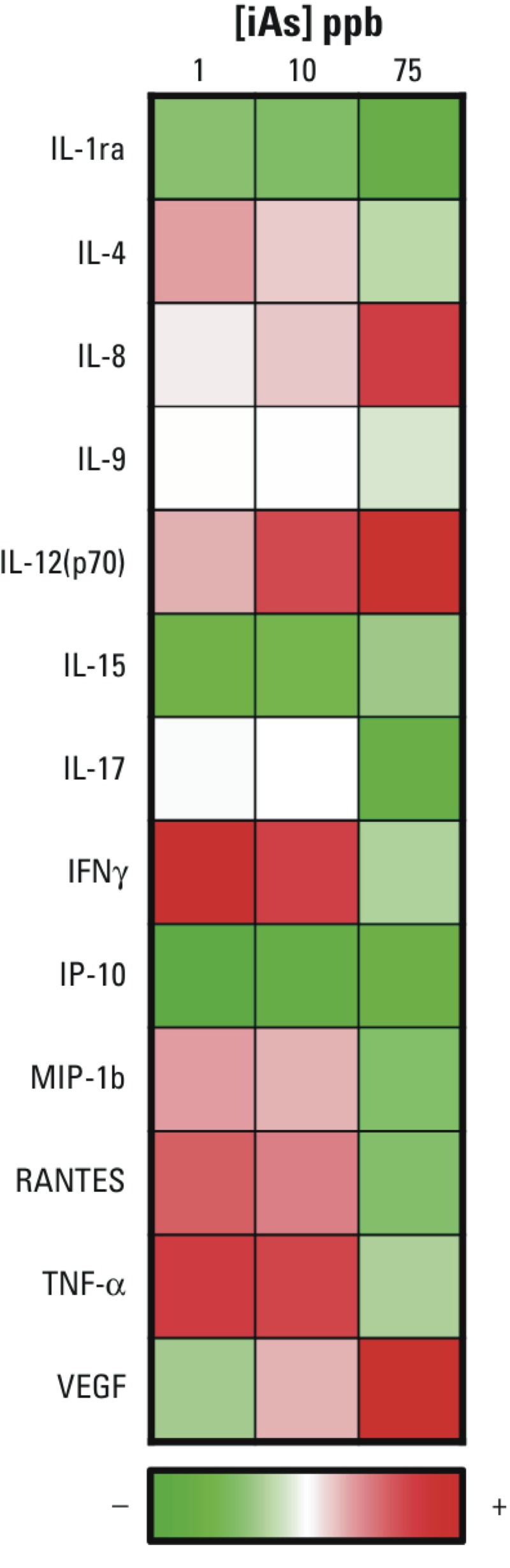
Concentration-dependent alteration of global cytokine profile in ASCs after iAs exposure. Results from the Bio-Plex Pro™ Human Cytokine 27-plex Assay (Bio-Rad) are displayed in a heat-map format (red = up-regulation, green = down-regulation). The average percent change of cytokine levels was determined by pooling adipose-derived mesenchymal/stromal cell (ASC) Donors 1 and 2 and taking the average level after 1 week exposure and comparing values for iAs-exposed ASCs with those of unexposed ASCs. The heat map shows the cytokines that had detectable levels and reveals a concentration-dependent expression profile.

Stromal–epithelial communication is known to be an important process in prostate cancer tumorigenesis and is mediated through soluble factors such as cytokines and growth factors (Barron and Rowley 2012). Based on the aforementioned coculture results that suggested iAs-exposed ASC CM enhanced prostate cancer cell viability, we next examined whether a potential mechanism for this observed change could involve disruption of the paracrine cytokine profiles of ASCs. We used a cytokine array to measure the average cytokine levels between both ASC donors in order to determine whether iAs mediated changes in their cytokine profiles. Figure 3 shows cytokines that met two major criteria: detectable levels of cytokine in the media and the presence of concentration-dependent alteration in cytokine levels with exposure. These criteria were used because we were particularly interested in iAs-induced changes and not necessarily in determining the basal cytokine levels. Cytokine levels were very similar between donors across the array with regard to the increase or decrease in cytokine level with increasing iAs dose; however, the magnitudes differed for some cytokines, once again suggesting some biological variance. When analyzing the mean cytokine levels, we observed the up-regulation of protumorigenic cytokines (VEGF and IL-8), whereas antitumorigenic cytokines (IL-1ra, IP-10, and IFNγ) were down-regulated (Figure 3). In addition, a concentration-dependent increase in IL-12 was observed with iAs exposure. These results suggest that iAs exposure may alter a wide range of cell–cell communication that could prime the stromal microenvironment to facilitate tumor progression.

### Proteomic Analysis of iAs-Exposed ASC

**Figure 4:**
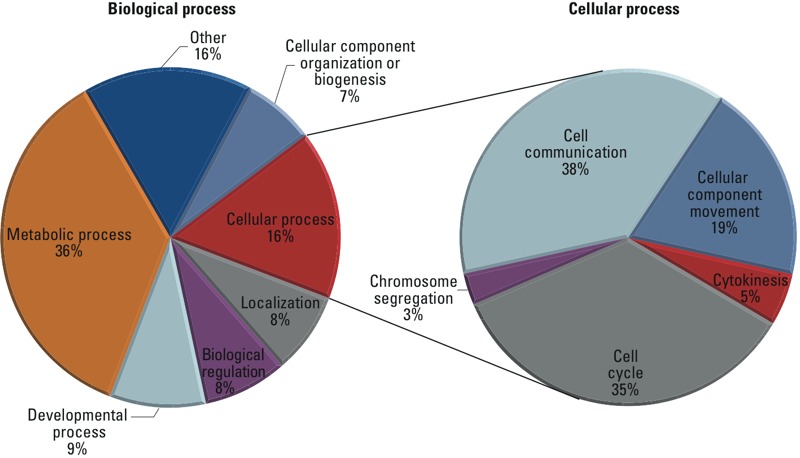
Proteomic analysis of iAs-induced changes in ASC signaling. Proteins determined by proteomic analysis to have statistically significant [*p* < 0.05, two-way analysis of variance (ANOVA)] differential expression after 1 week of exposure to 0 and 75 ppb inorganic arsenic (iAs) in both donors were uploaded into the online PANTHER GO Analysis program (http://www.pantherdb.org/).

To elucidate potential molecular mechanisms underlying the iAs-induced alteration of stromal cytokines, we performed a proteomic study of ASCs from two biological donors. ASCs from each donor were exposed to 0 or 75 ppb iAs for 1 week before proteomic analysis. We identified 1,114 unique proteins, of which 440 were significantly up-regulated and 170 were significantly down-regulated (*p* < 0.05) between 0- and 75-ppb samples in both ASC donors. Quantitative proteomic results were entered into PANTHER Gene Ontology (Mi et al. 2013) to ascertain which global pathways were dysregulated following iAs exposure. The PANTHER analysis is summarized in Figure 4. Interestingly, approximately 16% of identified proteins had functions related to cellular processes such as cell movement, cytokinesis, cell cycle, chromosomal segregation, and cell communication. These processes are interesting future avenues of research for investigating whether they are important stromal–epithelial signaling pathways disrupted during cancer progression following exposure to environmental toxicants. These results suggest that exposure to iAs over a short period of time may promote extensive rewiring of signaling in the stroma composing the tumor microenvironment.

### Potential Mechanism of iAs-Induced Perturbations in ASC

Proteomic analysis identified proteins associated with signaling pathways that potentially could underlie the paracrine effect mediated by iAs-treated ASC on prostate cancer viability. IPA was utilized to infer associations between significantly altered protein levels, including those of HMOX1 and THBS1. Both of these proteins have been associated with the TGFβ signaling pathway (Fitchev et al. 2010; Gueron et al. 2009; Okita et al. 2013). However, the mechanism interconnecting HMOX1 and THBS1 remains incompletely understood at present.

Proteomic anaylsis showed that the combined average expression (both ASC donors) of HMOX1 was up-regulated ~13-fold (*p* = 2.8 × 10^–6^) and that of THBS1 was down-regulated ~3-fold (*p* = 1.3 × 10^–6^). HMOX1, whose expression has been linked to arsenic-related oxidative stress response (Reichard et al. 2008), has a recently identified role in prostate tumor progression (Was et al. 2010). One of the major functions of THBS1 in the prostate is to convert latent TGFβ into its active form (Fitchev et al. 2010), but it can also serve as a potential biomarker to distinguish benign prostatic hyperplasia from malignant prostate cancer in epithelia (Shafer et al. 2007), suggesting its role in prostate cancer progression. Our data suggest that altered expression of these proteins may be associated with iAs exposure in ASCs.

**Figure 5:**
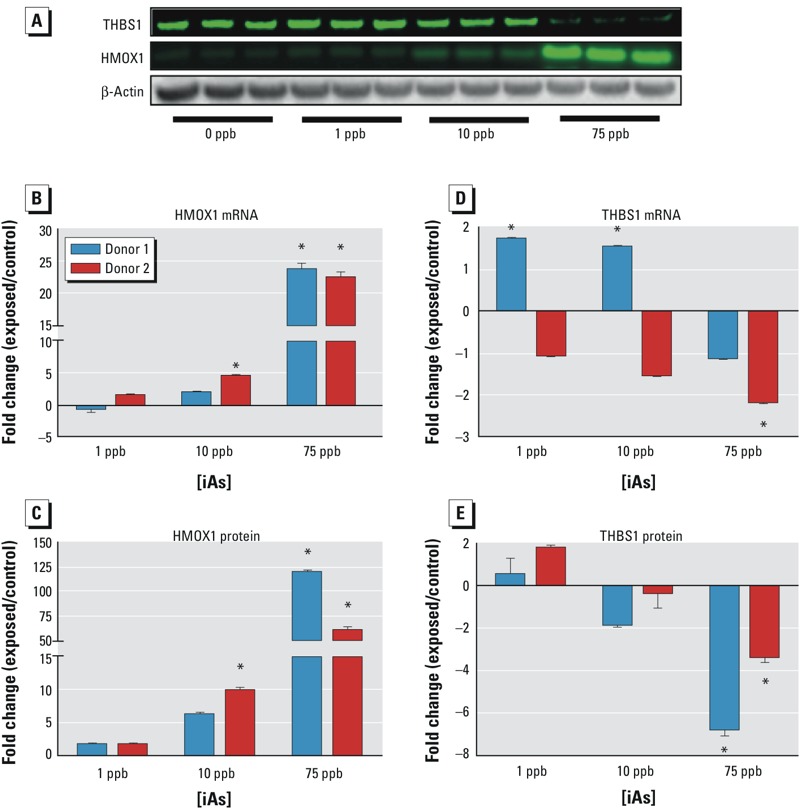
Validation of IPA-predicted HMOX1/THBS1/TGFβ Signaling Pathway. Adipose-derived mesenchymal/stromal cells (ASCs) were exposed to varying concentrations of inorganic arsenic (iAs) (0–75 ppb) for 1 week, and mRNA and protein levels were subsequently determined. (*A*) Representative Western blot; (*B*) HMOX1 mRNA expression; (*C*) HMOX1 protein expression; (*D*) THBS1 mRNA expression; (*E*) THBS1 protein expression. Expression is quantified as the mean ± SEM (*n* = 3) compared with a donor-specific unexposed sample. Statistical significance (*, *p* < 0.05 ) was calculated by two-way analysis of variance (ANOVA).

Focusing on these two candidate genes/proteins, we proceeded to validate the proteomic and IPA analyses through qRT-PCR and Western blot analysis. Figure 5A shows a representative Western blot for both proteins normalized to the housekeeping protein β-actin. Our results show a concentration-dependent increase in HMOX1 transcript and protein levels normalized to β-actin for both donors (Figure 5B,C) compared with donor-specific unexposed ASCs. Additionally, there appears to be an inverse relationship between iAs exposure and THBS1 transcript levels in both donors at a 75-ppb exposure (Figure 5D), whereas protein levels were significantly decreased in both donors at this level of iAs exposure (Figure 5E). Interestingly, Donor 1 had a statistically significant increase in THBS1 mRNA levels at exposures of both 1 and 10 ppb, which Donor 2 did not. Although this finding was statistically significant, it may not be biologically relevant because it was a relatively modest increase of < 2-fold. Additionally, it is possible that Donor 1 is simply not as sensitive to iAs exposure as is Donor 2, which our cytotoxic data corroborates (Figure 1) and may explain why discrepancies in mRNA expression were observed at the lower exposures. The protein level data, conversely, appeared to correlate better with statistical significance at the higher iAs exposures. These results validate the inferred IPA link between arsenite, HMOX1, THBS1, and the TGFβ pathway, particularly at the higher exposure levels. These data suggest a potential pathway that may be responsible for altering paracrine signaling between the stroma and tumor cell populations after exposure to iAs.

### TGFβ Signaling in iAs-Exposed ASCs

**Figure 6:**
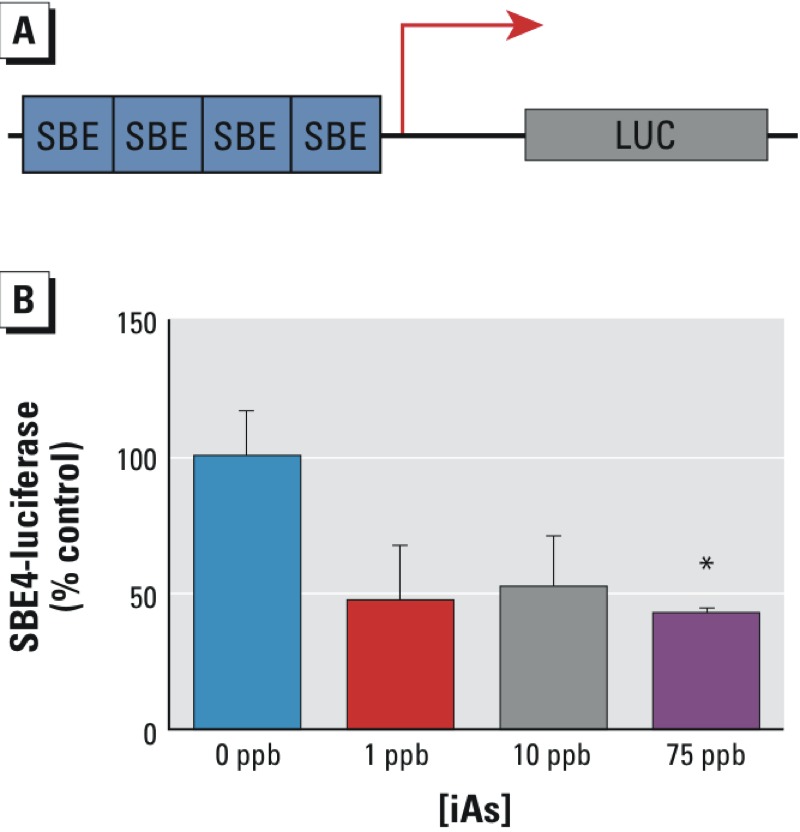
iAs decreases ASC TGFβ signaling. (*A*) Schematic representation of SBE4-luciferase signaling construct. (*B*) SBE4-luciferase signaling for adipose-derived mesenchymal/stromal cell (ASC) Donor 2 exposed to inorganic arsenic (iAs) for 48 hr. The results are displayed as the mean ± SEM (*n* = 3). Statistical significance (*, *p*-value < 0.05) was determined by one-way analysis of variance (ANOVA) comparing the 75-ppb exposure group with the other three exposure groups (0, 1, and 10 ppb).

TGFβ signaling is an important signaling pathway in tumorigenesis; TGFβ acts as both a tumor suppressor and promoter molecule depending on the microenvironment context (Principe et al. 2014). A decrease in stromal-mediated TGFβ signaling has recently been linked to increased malignancy in prostate cancer (Li et al. 2008). This finding led us to examine whether iAs exposure was capable of attenuating stromal TGFβ signaling as inferred by IPA. We transfected ASCs with a luciferase construct that is a reporter of TGFβ pathway activity because it contains four binding sites for phosphorylated SMAD2/3/4 regulators (SBE4-Luc, Figure 6A) (Zawel et al. 1998). Unexposed and iAs-exposed ASCs were subsequently monitored for luciferase levels following 48 hr of exposure. Donor 2 was selected for luciferase reporter assay analysis owing to the higher transfection efficiency for cells from this donor. The results from our luciferase reporter assay suggested that exposure to iAs (1–75 ppb) attenuated TGFβ signaling in ASCs, with levels of down-regulation ranging from ~43% to 52% compared with levels in unexposed ASCs (Figure 6B). Of all the groups, only ASCs exposed to 75 ppb iAs showed statistically significant differences when the groups were compared using one-way ANOVA.

## Discussion

The ramifications of stromal signaling dysregulation in the context of cancer progression are still being unraveled. In prostate cancer, for instance, the tumor microenvironment has been identified as an important factor associated with cancer progression (Barron and Rowley 2012). To the best of our knowledge, no previous reports have examined the effects of environmental contaminants on stromal cells or the ability of iAs exposure to mediate protumorigenic effects by affecting the stroma.

Attenuation of TGFβ signaling in the stroma has recently been shown to be a critical player in the formation of reactive stroma (Banerjee et al. 2014). However, until now, there has been little direct evidence for the promotion of an adverse stromal cell reaction by environmental toxicants in an analogous manner. Here, we demonstrate that the environmental contaminant iAs can alter global signals mediated by stromal cells, potentially through down-regulation of TGFβ signaling. This effect has important implications in that a more reactive stroma might develop after iAs exposure, leading to enhanced prostate cancer progression.

One of the hallmarks of cancer progression is the uncontrolled proliferation of tumor cells. Stromal cells can contribute paracrine signals (growth factors) that can strongly influence the growth of surrounding epithelial cells (Witz 2008). Using a conditioned-media coculture model, we have demonstrated that iAs has the potential to alter the secretory signature of stromal cells by modifying the proteomic and expression profiles of ASCs. Here, iAs significantly modified ASCs towards a signaling signature consistent with stimulation of prostate cancer cell growth. The main implication of these findings is that environmental toxicants can affect the stromal compartment to augment tumor promotion, particularly in the context of inflammation.

To better understand the effects of iAs-treated ASCs on enhancing prostate tumor cell viability mediated through paracrine interactions, we investigated a panel of alterations using a cytokine array. ASCs are enriched in the prostate microenvironment of prostate cancer patients (Ribeiro et al. 2012c). Ribeiro et al. suggested that prostate cancer progression is facilitated by stromal-mediated signaling, which increases hypercellularity and decreases antitumor immunity (Ribeiro et al. 2012a). In line with Ribeiro’s evidence, we found a concentration-dependent relationship with an increase of protumorigenic cytokines (IL-8 and VEGF) and a decrease in antitumorigenic cytokines (IL1Ra, IP10, and IFN-γ) following exposure to iAs. Stromal IL-8 expression has been linked to prostate cancer growth and metastatic ability (Thorpe 2013). Although substantial evidence has suggested a role for VEGF in prostate cancer progression (Roberts et al. 2013), only recently has its stromal expression been implicated as well (Ding et al. 2013).

An increase in the stromal contribution of protumorigenic cytokines and simultaneous concentration-dependent decreases in the antitumorigenic cytokines IL-1Ra, IP-10, and IFN-γ expression were identified. Expression of IL-1Ra (Voronov et al. 2003), IP-10 (Nagpal et al. 2006), and IFN- γ (Hastie et al. 2008) has been linked to a decrease in prostate cancer severity. Interestingly, the concentration-dependent increase of the proinflammatory cytokine IL-12 suggests an important role for immune system activation in the tumor microenvironment because IL-12 production has been shown to be important in T-cell maturation (Manetti et al. 1993), meriting further studies *in vivo* in the context of a competent immune system. These results suggest that iAs can modulate changes in the tumor microenvironment by altering cell–cell communication, which in turn is responsible for balancing pro- and antitumorigenic signals.

Additionally, we have described a novel mechanism of action of iAs that may be associated with stromal–epithelial communication, which may involve the HMOX1/THBS1/TGFβ signaling axis. HMOX1 expression has previously been shown to be an indicator of arsenic-induced oxidative stress in cells (Reichard et al. 2008). However, a recent review highlighted a different function for HMOX1 in tumor progression (Was et al. 2010). Activation of HMOX1 as a nuclear receptor has been linked to increased VEGF expression, which may constitute an important link between some of the global cytokine changes detected in our study and HMOX1 expression (Gueron et al. 2009). However, HMOX1 expression has also been described as being inversely related to THBS1 expression (Gueron et al. 2009). THBS1 is an antitumorigenic factor essential for normal prostate angiogenesis whose loss has been linked to both tumor progression and enhanced VEGF expression (Doll et al. 2001). THBS1 has also been shown to be essential for the conversion of latent TGFβ into its active form, which is required to initiate the subsequent signaling cascade (Fitchev et al. 2010). Because TGFβ is a soluble paracrine factor, the potential loss of activation by a decrease in stromal THBS1 levels may result in a decrease in stromal-derived TGFβ concentration in the microenvironment niche, potentially explaining the decrease in TGFβ signaling. Disruption of stromal TGFβ signals in prostate cancer has already been identified as an important mediator of prostate cancer progression in the context of bone lesions (Li et al. 2012). This information, combined with a recent report suggesting alterations to TGFβ signaling in stroma as a causative agent of epithelial lesions (Banerjee et al 2014), suggests that the TGFβ pathway must be tightly regulated in stroma in order to maintain epithelial tissue integrity. Interestingly, we have shown that an environmental contaminant can induce effects on the stroma similar to those achieved by the genetic ablation of the TGFβ receptor by potentially attenuating TGFβ signaling. Our findings suggest that environmental contaminants have the potential to create an environment suitable for the progression of cancer within the stroma, predisposing the epithelia to tumorigenesis. This mechanism could eventually have important therapeutic implications by targeting the stromal compartment of the tumor microenvironment.

## Conclusions

Our results illustrate the biochemical complexity associated with iAs carcinogenesis. We demonstrated the capability of iAs to remodel the topology of ASC signaling, potentially modulating a variety of processes such as cell proliferation, angiogenesis, and immune surveillance in the tumor microenvironment. Our findings support the hypothesis that iAs exposure affects not only existing tumors but also can affect the environment surrounding epithelial cells, which may increase the predisposition for tumor formation. By better understanding how the stromal microenvironment can be modulated by environmental stressors, we may gain better insight into the etiology of cancer. This insight could eventually lead to the development of adjuvant strategies aimed at targeting the tumor stroma, which may improve current interventions that suffer from a lack of efficacy.
